# Improvement to ultrasonographical differential diagnosis of gastric lesions: The value of contrast enhanced sonography with gastric distention

**DOI:** 10.1371/journal.pone.0182332

**Published:** 2017-08-04

**Authors:** Tingting Li, Man Lu, Jun Song, Ping Wu, Xueqing Cheng, Zhenqi Zhang

**Affiliations:** 1 Department of Ultrasound, Sichuan Cancer Hospital Institute, Sichuan Cancer Center, School of Medicine, University of Electronic Science and Technology of China, Chengdu, China; 2 Department of Ultrasound, Sichuan Academy of Medical Sciences & Sichuan Provincial People’s Hospital, School of Medicine, University of Electronic Science and Technology of China, Chengdu, China; Shanghai Tenth People's Hospital, CHINA

## Abstract

**Objective:**

The purpose of this retrospective study is to evaluate the diagnostic value of contrast enhanced sonography plus gastric distention sonography, the Double Contrast-enhanced Ultrasound (DCUS) in gastric lesions.

**Methods:**

107 cases with pathology confirmed gastric lesions were retrospectively reviewed, DCUS and oral contrast agent ultrasound (US) were performed in all cases prior to operation. Perfusion parameters including arrival time (AT), peak intensity (PI), time to peak (TTP), and area under the curve (AUC) of the lesion and surrounding normal tissue were analyzed. A reader blinded to pathology results were asked to rate and compare each case with surgical or resection biopsy pathology results.

**Results:**

From the 107 gastric lesions, 75 were malignant gastric lesions (33 gastric cancers,42 gastrointestinal stromal tumors (GISTs)) and 32 were benign gastric lesions (11 inflammatory masses and 21 polypoid adenomas). Compared with US, DCUS achieved higher value in sensitivity (90.6% vs. 70.6%), specificity (75% vs. 62.5%), positive predictive value (89.5% vs. 81.5%), negative predictive value (77.4% vs. 47.6%), and overall accuracy (85.9% vs. 68.2%). When US was tested against DCUS, the increase in correct diagnoses value was significant (P = .01). Furthermore, gastric cancer had faster AT, higher PI and AUC than normal tissue (P<0.05); GIST and Inflammatory mass had higher PI than normal tissue (P<0.05); gastric cancer and GIST had faster AT than polypoid adenoma (P<0.05), Inflammatory mass showed higher PI than other 3 lesions and gastric cancer had higher PI than polypoid adenoma and GIST (P<0.05); gastric cancer and inflammatory mass had larger AUC than polypoid adenoma and GIST (P<0.05). Conclusion DCUS improved diagnostic performance compared with US. The combination of different CEUS enhancement characteristics with quantitative perfusion parameters may provide a promising tool to help differentiate gastric cancer and GIST from benign lesions.

## Introduction

Gastric lesions encompass a variety of non−neoplastic and neoplastic conditions (both benign and malignant). As gastric cancer is the second leading cause of cancer morbidity and mortality in China [1], differentiating malignant from benign gastric lesions is particularly important. Gastric lesions were detected by various imaging modalities, including barium radiography, endoscopy, endoscopic ultrasonography (EUS), CT and MRI; Several studies showed that EUS is the best choice in diagnosing gastric lesions, particularly submucosal lesions, which is better than CT and endoscopy [2,3]. Joo Ha. et al reported, however, that EUS morphological diagnosis is correct in only 48% of their cases and EUS-FNA examination is essential. Biopsy sampling from submucosal lesions often remains difficult and increases the risk of bleeding and perforation [4]. Indeed, in the previous study, they suggested that EUS-FNA may increase the risk of tumor seeding after biopsy [5]. Furthermore, it is adequate for those asymptomatic patients with benign lesions to be followed and observed rather than undergo surgical therapy.

Conventional trans-abdominal ultrasonography plays a minor role in the routine examination of the luminal gastrointestinal tract. However, trans-abdominal sonography with oral contrast agent (US) can depict the 5-layer pattern of gastric wall as well as EUS and optimize morphologic assessment. Several studies have demonstrated the utility of oral contrast agent in evaluating digestive diseases [6,7]. Contrast-enhanced ultrasound applications in solid organs such as liver, kidney and spleen have been widely used and well accepted in many countries. The intravenous contrast medium accentuates the perfusion pattern in tissues to help differentiate different types of lesions within the organ.

Double contrast-enhanced ultrasound (DCUS) combines both the oral contrast agent with intravenous contrast, and was developed as a novel method to detect digestive tract diseases such as gastric cancer and rectal lesions in China [7–9]. However, the diagnostic value of DCUS for malignant and benign gastric lesions remain unclear. Furthermore, differential diagnosis of various gastric lesions by combining different CEUS enhancement characteristics with quantitative perfusion parameters also need to be explored. In this single center study, we evaluate the diagnostic value of DCUS and its ability in recognizing various gastric lesions by testing against surgical pathology results.

## Patients and methods

### Patients

From December 2013 to November 2015, a total of 107 consecutive gastric lesions were enrolled in this study. The inclusion criteria were as follows: (1) gastric lesions should be certificated by surgery or excision biopsy by pathology confirmation. (2) suspected lesions underwent the CEUS examination. (3) all selected lesions were primary disease; The exclusion criteria were as follows: (1) unsatisfactory DCUS image quality for quantitative analysis or incomplete data. (2) absence of pathological diagnosis as the gold standard. (3) lesion caused by metastasis from other organs. (4) patients with gastrointestinal surgical history; All patients provided signed informed consent before the DCUS examination. The clinical trial was approved by the Institutional Review Board and Ethics Committee of Sichuan Cancer Hospital.

### Equipment

The DCUS examinations were completed with a Philips iU22 and iU Elite system (Philips Medical Systems, Bothell, WA), which was equipped with contrast pulse sequencing technology, and a computer equipped with an off-line Q-lab quantitative analysis software (Philips Medical Systems, Bothell, WA). An abdominal transducer C5-1 (convex bandwidth, 1–8 MHz) was used for imaging, using a low–mechanical index and contrast mode of sonography.

The oral contrast agent was a powder made up of soybean and rice (48g/package, TianXia, Huzhou, Zhejiang, China). Each package of the agent was diluted with 500ml boiling water and gently mixed to a homogeneous suspension. The suspension was then cooled to 35–45 centigrade for ingestion by the patient. The intravenous contrast agent used was SonoVue (BraccoSpA, Milan, Italy), made up of sulfur hexafluoride (SF6)-filled microbubbles.

### Procedure

#### 2D-ultrasonography

All patients were asked to fast at least 8 hours before the examination. Patients ingested 500ml of the oral contrast agent which fills the stomach. By using a continuous multi-directional (longitudinally and transversely) scanning method, we examined the cardia, fundus, pylorus and duodenal bulb dynamically in supine, left and right lateral decubitus positions. The normal 5-layer of normal gastric wall was identified during the examination followed by assessment of the target lesion.

#### CEUS

After the morphological assessment (including shape, location and the size), a 2.4ml bolus of Sonovue was injected intravenously, followed by a 5-mL saline flush. Immediately thereafter real-time contrast imaging was recorded continuously for at least 5 minutes. Patients were kindly asked to breathe slowly and superficially. No hypotonizing or sedating agent was administered. All DCUS was performed and recorded by a gastric ultrasound specialist (Man Lu) who was blinded to the endoscopy and other radiography results. According to the comparison of different enhancement intensity between lesion and surrounding normal gastric wall, all the lesions were categorized to hypo-enhanced, unenhanced, and hyper-enhanced pattern by operator.

#### Perfusion index

Image Analysis

The cine loops were reviewed and interpreted independently by 2 radiologists (J.S. and P.W.) who were blinded to the pathological results and the initial ultrasonographer. The pathological standards include biopsy and resection results. By using the Q-lab quantitative analysis software, the region of interest (ROI) in each patient was selected with the same area for both the lesion in question and surrounding normal tissue. The parameters of CEUS including arrival time (AT), peak intensity (PI), area under the curve (AUC), time to peak (TTP) were obtained and recorded by the auto-tracking contrast quantification software. To avoid motion artifact caused by breathing, Image Motion Compensation was turned on and ROIs were adjusted manually from frame to frame.

#### Statistical analysis

Data was analyzed using SPSS 19.0 (IBM Corporation, Armonk, NY).All CEUS parameters and patient characteristics were expressed as mean ± standard deviation (mean ± SD). By testing against pathological result as reference standard, the diagnostic sensitivity, specificity, positive predictive value (PPV), negative predictive value (NPV), and accuracy of US and DCUS for differentiation between benign and malignant gastric lesions were calculated based on every lesion, the diagnostic differences between US and DCUS were analyzed with Chi-square test. The differences between different lesions and its neighboring normal tissue were compared by Paired-sample T-tests, and one-way ANOVA was used to compare the difference of each CEUS parameters among the 4 different types of gastric lesions. P<0 .05 was considered statistically significant in all analyses.

## Results

### Demographics

A total of 103 patients were recruited in this study, and 107 lesions were assessed in the final analysis (4 patients with double lesions). The mean size was 2.72±1.14cm (range 1-5cm). Pylorus and fundus were the location where most lesions situated. The pathological diagnoses of these 107 lesions included: 33 gastric cancers (23 adenocarcinomas, 4 signet-ring cell carcinomas, and 6 neuroendocrine carcinomas (NETs)), 42 GISTs (13 high-risk tumors, 6 middle-risk tumors and 23 low-risk tumors), 11 inflammatory masses, and 21 polypoid adenomas (Table 1) S1 Table.

**Table 1:** Patients characteristics.

**Age, y, mean ± SD**	62.7±11.8
**Size,cm, mean ± SD**	2.72±1.14
**Gender,male/female**	61/42
**Pathology,n**	107
**Gastric cancer,total**	33
**adenocarcinoma**	23
**Signet ring carcinoma**	4
**Neuroendocrin carcinoma**	6
**Inflammatory Mass**	11
**Polypoid Adenoma**	21
**GIST, total**	42
**Low risk**	23
**Moderate risk**	6
**High risk**	13

### Accuracy

The sensitivity, specificity, PPV, NPV, and overall accuracy of US in differentiation between benign and malignant gastric lesion were 70.6%, 62.5%, 81.5%, 47.6% and 68.2% versus 90.6%, 75%, 89.5%, 77.4% and 85.9% for DCUS, respectively. The increase in correct diagnoses was significant (P < .01) when US was compared against DCUS (Table 2).

**Table 2:** The sensitivity, specificity, PPV, NPV, and overall accuracy of gastric distention US and DCUS.

	Sen(%)	Spe(%)	PPV(%)	NPV(%)	Accuracy (%)
**US**	70.6	62.5	81.5	47.6	68.2
**DCUS**	90.6	75	89.5	77.4	85.9

**Abbreviation:** Sen (%) (sensitivity), Spe (%) (specificity), PPV (%) (positive predictive value)

NPV (%) (negative predictive value)

### DCUS Features

#### Gastric cancer

**Fig 1:**
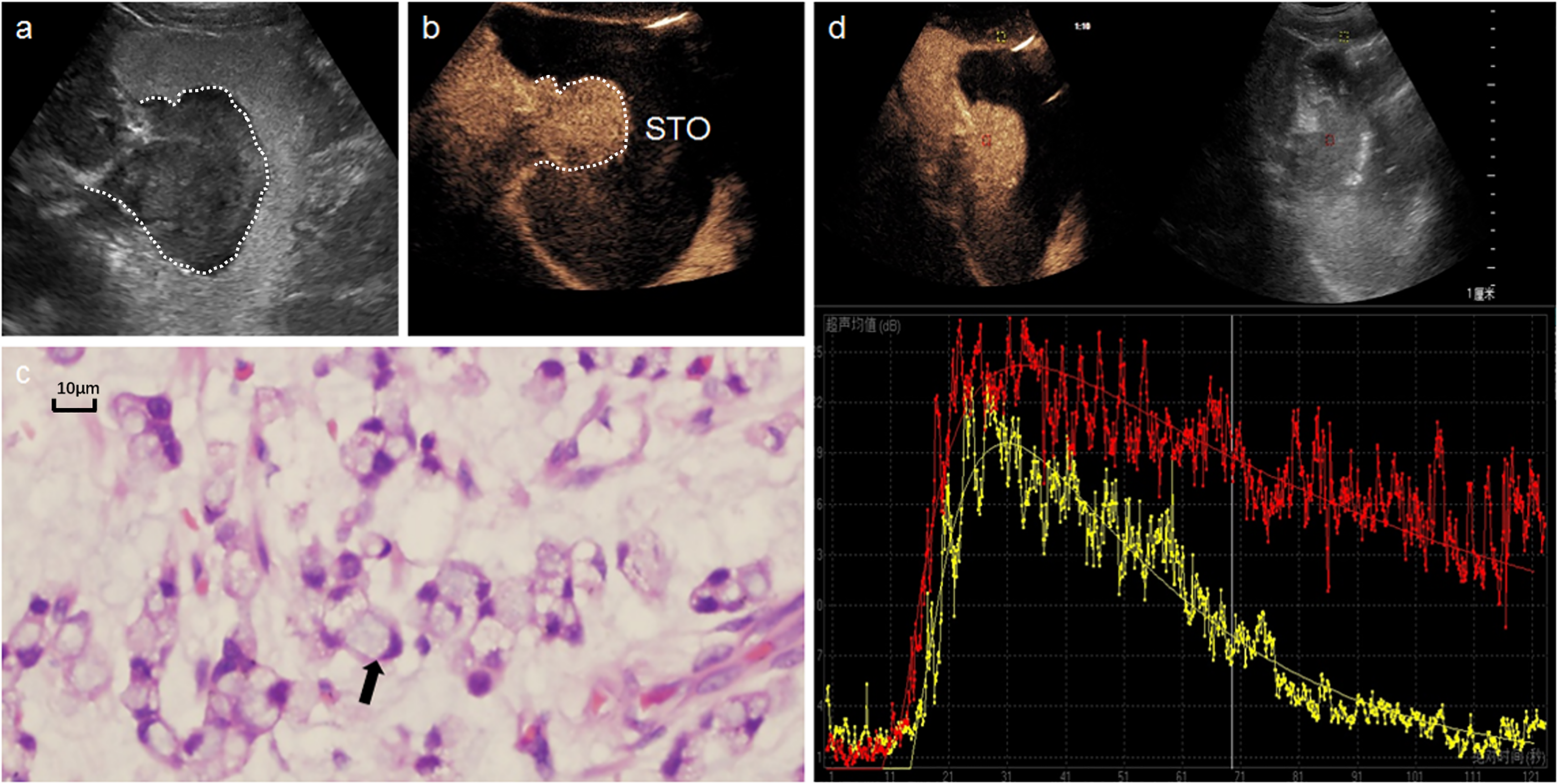
A 65-year-old man with a gastric signet-ring cell carcinoma. **a:** Oral contrast agent ultrasound detected an irregular mass in gastric fundus. **b:** DCUS showed a homogeneous hyper-enhancement tumor. **c:** Many signet-ring cells (hematoxylin and eosin, × 200) in microscopic field. **d:** Time-intensity curve depicted the lesion (red line) have faster AT, higher PI and AUC than surrounding normal tissue (yellow).

Of the 33 gastric cancers, the gastric walls were thickened focally and hypoechoic lesions were protruding into the lumen.20/33 lesions had irregular surface with a hyperechoic plaque. Among the DCUS characteristics of gastric cancer, there were 15/33 lesions showing homogeneous hyper-enhancement, 9/33 lesions showed heterogeneous hyper-enhancement, 5/33 lesions showed homogeneous hypo-enhancement, and 4/33 showed heterogeneous hypo-enhancement. Gastric cancer had a significantly faster AT, higher PI and AUC than surrounding normal tissue (P<0.05). (Fig 1; Table 3)

**Table 3:** CEUS parameters of different gastric lesions and compare with normal tissue.

Lesions	AT(s)	PI(dB)	AUC(dB s)	TTP(s)
**gastric cancer**	6.9±1.5	18.1±3.6	1056.7±456.2	30.7±7.9
**normal tissue**	12.5±3.8	12.1±3.0	622.9±241.6	36.3±16.7
***P***	<0.001*	<0.001*	<0.001*	>0.1
**GIST**	8.9±4.9	13.4±4.3	489.1±300.5	26.9±11.2
**normal tissue**	11.7±6.7	11.5±3.3	429.6±244.1	19.8±10.5
***P***	0.08	0.04*	>0.1	>0.1
**inflammatory mass**	8.9±3.2	20.8±4.3	1270.7±583.8	28.6±6.7
**normal tissue**	11.8±4.7	15.3±3.0	953.9±337.7	34.6±12.1
***P***	>0.1	<0.001*	0.06	0.07
**polypoid adenoma**	12.1±3.9	13.3±3.7	511.2±271.6	27.1±5.4
**normal tissue**	11.7±4.4	12.4±6.3	486.8±336.5	32.9±12.7
***P***	>0.1	>0.1	>0.1	0.07

**Abbreviation:** AT (arrival time), PI (peak intensity), AUC (area under the curve)

*P< .05

#### GISTs

**Fig 2:**
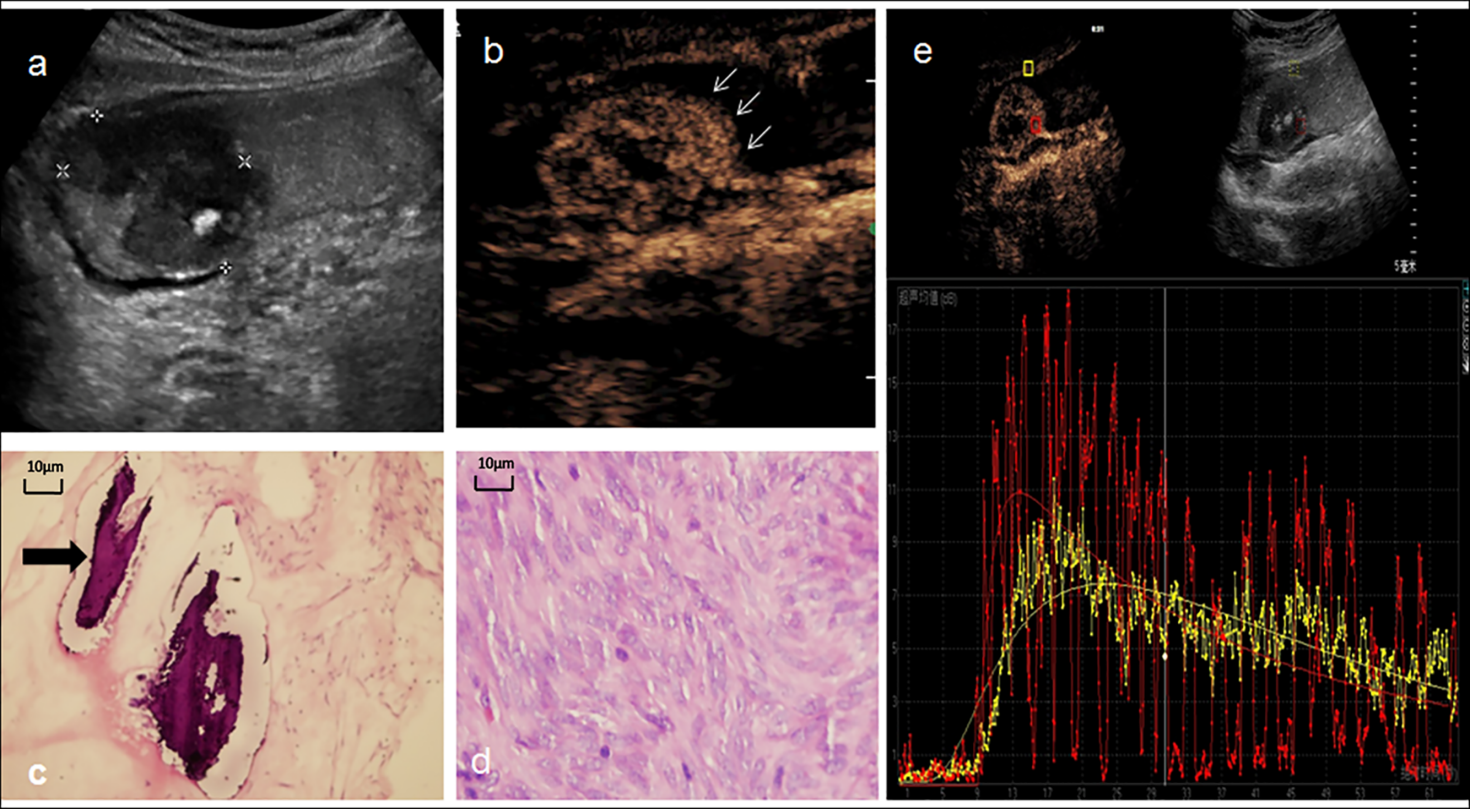
A 43-year-old woman with a gastrointestinal stromal tumor. **a:** Conventional trans-abdominal ultrasound with oral contrast agent showed a lobulated heterogeneous hypoechoic lesion (calipers) located in antrum of stomach, a hyper-echoic calcification plaque could be detected inside of the tumor. **b:** DCUS showed a ring-like hyper-enhancement (arrow) and heterogeneous hyper-enhanced inside of the lesion. **c,d:** Microscopic certificated the calcification in GIST (arrow in c)and fascicular proliferation of spindle-shaped cells.(d)(hematoxylin and eosin, × 200). **e:** Time-intensity curve depicted the lesion (red line) have higher PI and faster AT, TTP than surrounding tissue (yellow), final pathology result classified this GIST into high-risk category.

Gastrointestinal stromal tumors appeared as a round, oval or lobulated shape with homogeneous hypoechoic or heterogeneous hypoechoic pattern, likely due to necrosis caused by their large size, and calcification plaques in the lesions. After DCUS, 35/42 GISTs showed a ring like hyper-enhancement. Centrally within the lesion, there were 15/42 demonstrating homogeneous hypo-enhancement, 8/42 lesions heterogeneous hypo-enhancement, 12/42 homogeneous hyper-enhancement, and 7/42 heterogeneous hyper-enhancement. GIST had a significantly higher PI compared to neighboring normal tissue (P<0.05) (Fig 2; Table 3)

#### Inflammatory mass

**Fig 3:**
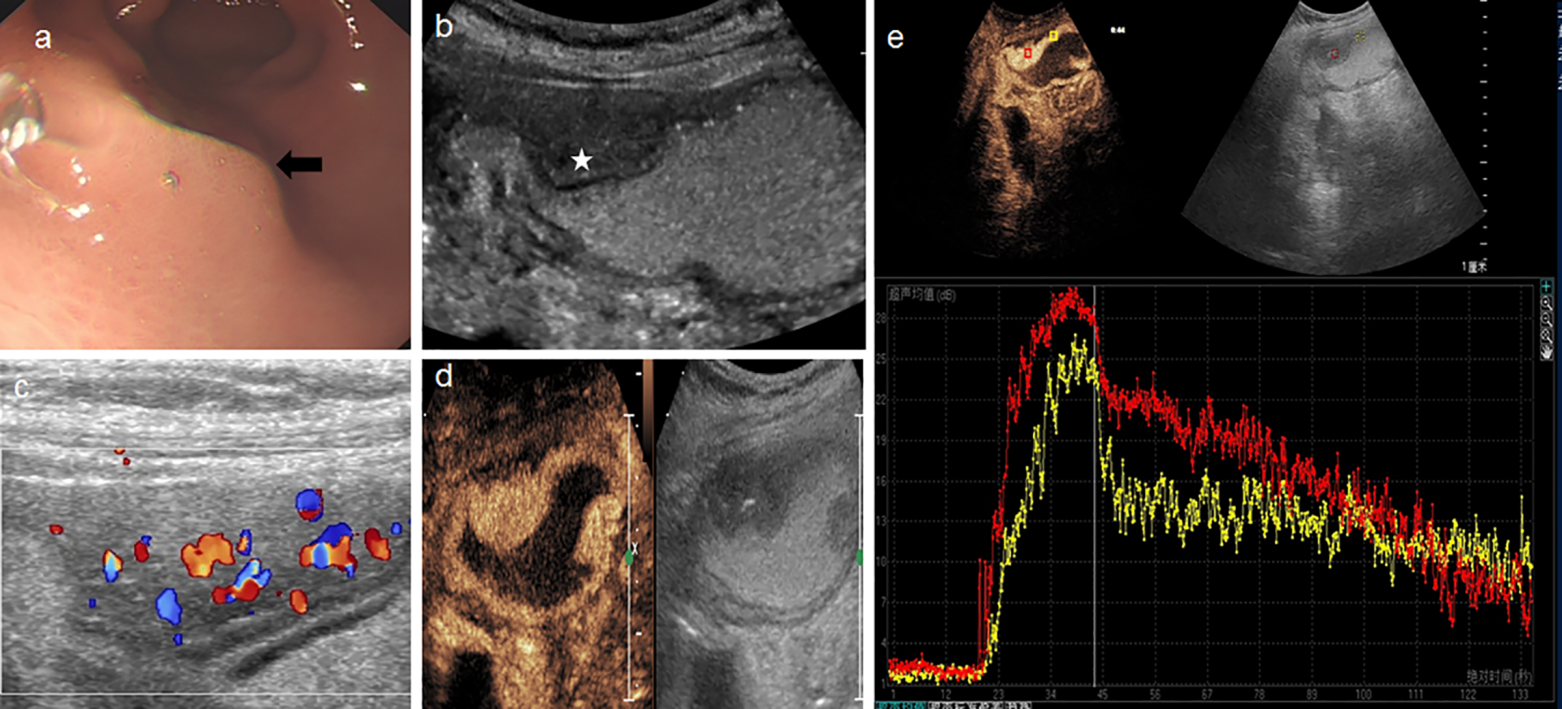
A 59-year-old woman with an inflammatory mass. **a:** Gastro-endoscope detected a bump with intact overlying mucosa protruding into the cavity. **b:** Conventional trans-abdominal ultrasound with oral contrast agent showed a well-defined hypoechoic lesion (star), with an intact mucosa. **c:** multiple vessels were visible on the color Doppler. **d:** DCUS showed a homogeneous hyper-enhanced mass in the antrum. **e:** Time-intensity curve depicted the lesion (red line) have higher PI than surrounding normal tissue (yellow).

The thickened mucosal layer in gastric inflammatory lesions showed a mass appearance protruding into the cavity with a homogeneous echotexture. After CEUS, 6/11 lesions showed homogeneous hyper-enhancement, and the other 5/11 lesions showed heterogeneous hyper-enhancement. Inflammatory masses had a significantly higher PI than normal tissue (P<0.05) (Fig 3; Table 3)

#### Polypoid adenomas

**Fig 4:**
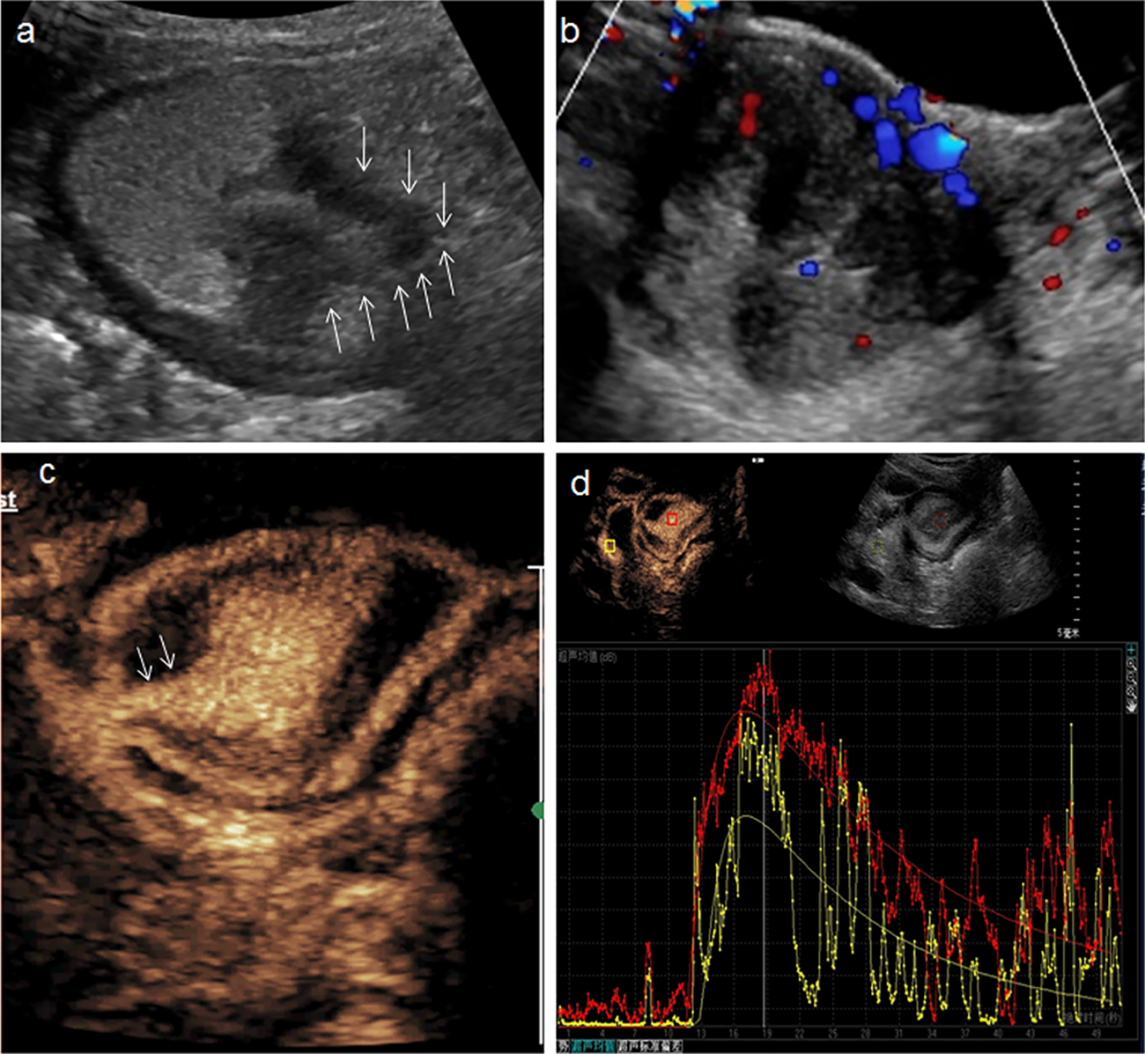
A 65-year-old man with a polypoid adenoma. **a:** Conventional trans-abdominal ultrasound with oral contrast agent showed an irregular hypoechoic lesion (arrow) located in antrum. **b:** Multiple flow signals were visible on the color Doppler. **c:** DCUS showed homogeneous hyper-enhanced mass connected with gastric wall by a pedicle (arrow). **d:** Time-intensity curve depicted the lesion (red line) have higher PI than surrounding normal tissue (yellow).

Polypoid adenomas always originated from mucosal layer and formed a mass which had the morphology of a water-droplet, or beansprout if the lesion had a pedicle connected with gastric wall (Fig 4). 9/21 polypoid adenomas demonstrated heterogeneous hyper-enhancement and the rest of them showed homogeneous hyper-enhancement. None of the CEUS parameters showed statistical significance between polypoid adenomas and normal tissue. (Fig 4; Table 3)

Further analysis of CEUS parameters of the 4 different types of lesion was completed to determine if any feature is associated with a particular diagnosis. Gastric cancer and GIST had a faster AT than polypoid adenoma (P<0.05). Inflammatory mass showed the highest PI, followed by gastric cancer, polypoid adenoma, and GIST (P<0.05). Gastric cancer and inflammatory mass had a significantly larger AUC than polypoid adenoma and GIST (P<0.05). (Table 4)

**Table 4:** Comparison of CEUS parameters between the different gastric lesions (P value).

	AT	PI	AUC	TTP
**gastric cancer VS GIST**	>0.1	<0.001*	<0.001*	>0.1
**gastric cancer VS inflammatory mass**	>0.1	0.02	>0.1	>0.1
**gastric cancer VS polypoid adenoma**	<0.001*	<0.001*	<0.001*	>0.1
**inflammatory mass VS GIST**	>0.1	<0.001*	<0.001*	>0.1
**polypoid adenoma VS GIST**	0.03*	>0.1	>0.1	>0.1
**inflammatory mass VS polypoid adenoma**	>0.1	<0.001*	<0.001*	>0.1

**Abbreviation:** AT (arrival time), PI (peak intensity), AUC (area under the curve)

*P< .05

## Discussion

Trans-abdominal sonography with oral contrast agent and Double Contrast-enhanced Ultrasound (DCUS) have been widely used in China, but their diagnostic value for differentiating malignant and benign gastric lesions remain unclear. Gastric distention sonography, in view of its convenience and cost-effectiveness, especially in screening for malignant gastric lesions, such as gastric cancer, showed a 77.3% accuracy in determining T stage of gastric cancer [6]. In this differential diagnosis study, we reported the accuracy, sensitivity and specificity were 68.2%,70.6% and 62.5% respectively in gastric distention ultrasonography, they were all lower than previous study, this discrepancy may have been related with the higher age structure of our patient pool, which have a mean age were 62.7 years old and this flatulence influenced our results. However, within the same condition, DCUS had a higher sensitivity, specificity in 90.6%, 75.0% respectively; Recently, DCUS were mainly reported in tumor staging and predicting lymph node status in gastric cancer, with sensitivity varying from 78% to 92%, specificity ranging from 60.7% to 88.0% [10–12]. Nevertheless, in our study, the results suggested that DCUS increased diagnostic accuracy in differential benign and malignant gastric lesions, which, to our knowledge, is this first report in literature.

In this study, transabdominal sonography with oral contrast agent could clearly display the 5-layer of normal gastric wall and determine the origin layer and relevant morphological information of the lesion, such as calcification and necrosis. Oral contrast agent sonography can easily determine extraluminal compression, which may be confused with submucosal lesions in endoscopy examinations. In our study, an endoscopically suspected gastric lesion was excluded because gastric-distension sonography proved the lesion was the normal fundus of the gallbladder. For malignant lesions, metastatic lymph nodes could also be detected during routine examinations.

Contrast-enhanced ultrasound (CEUS) is a safe, non-invasive examination which shows the blood flow and vascular perfusion to target tissue, this technique has been widely used to assess lesions in the liver, kidney, and breast and can provide accurate information comparable to CT and MRI [13]. The combination of oral and intravenous contrast, not only could give the same details of the lesion such as size, shape, contour and echogenicity as EUS, but also improves the ability to distinguish gastric cancer and GIST from benign lesions noninvasively by CEUS analysis. In our study, most gastric cancers were hyper-enhancing, but almost one third (10/33) showed hypo-enhancing, 6 of them being neuroendocrine tumors. Malignant risk varied in different histotypes, but angiogenesis is essential for their future growth, invasion, and metastasis [14]. With regards to other malignant lesions, GISTs could be classified into low risk, intermediate risk, and high risk for malignant potential [15]. The origin layer of most GISTs is within the muscularis propria and gradually grow into the lumen. This may give a qualified explanation why 35 in 42 GISTs showed a ring-like hyper-enhancement but varied centrally within the tumor. Inside of the GISTs, they may have necrosis, calcification, hemorrhage or cyst formation because of different size and degree of malignant potential, coincidence with the DCUS appearance [16,17]. According to the study, only patients with large NETs (>1cm) and large GISTs (>2cm) should be locally resected, while asymptomatic patients with small tumors should undergo surveillance [18]. DCUS is a promising modality to follow up in this patient pool, in particular for the elderly population which invasive endoscopic surveillance carries risks.

In addition, DCUS in our study provided perfusion index and perfusion pattern to identify different lesions. According to our quantitative analysis of contrast indexes, gastric cancer and inflammatory masses had higher PI than normal tissue in this study, likely as a result of neovascularization. Eleven inflammatory lesions in our study showed thickened but still flexible gastric wall and were significantly different than gastric cancers, which were appeared as stiff wall and mostly hyper-enhancing. It is worth noting that GIST had higher PI than surrounding normal gastric wall but lower than gastric cancer and inflammatory mass, this result may correspond with low-risk tumors, which accounted for most in our GIST cases.

Furthermore, the following experience that we summarized during the examination was also useful. For 5 hypoechoic masses which were suspected GISTs, DCUS demonstrated that the lesions were hyper-enhanced circularly but non-enhanced inside of the tumor. These “pseudo-GISTs” were determined to be cystic lesions and under surveillance in our department. DCUS can therefore be helpful in differentiating GIST and gastric cysts. DCUS could also be a novel method to cover the shortage that some polypoids were compressed by the impregnate oral contrast agent and increase the detection rate of small polypoids. A ring-like hyper-enhancement mass with necrosis and calcification within the tumor likely indicates a GIST, and may reduce the need for a EUS FNA.

There are several limitations in the current study. First, some types of benign submucosal lesions were not included due to insufficient cases or missing pathological confirmation, such as ectopic pancreas, lipomas, and leiomyomas. Secondly, due to insufficient number of partial cases, we didn’t evaluate the diagnostic accuracy by each pathological type. A larger variety and number of cases is needed to determine the DCUS characteristics of more rare lesions.

## Conclusion

In conclusion, Double contrast ultrasound characteristics with quantitative perfusion parameters may provide a promising tool to help differentiate gastric cancer and GIST from inflammatory lesions or polypoid adenomas. DCUS is a convenient, non-invasive, non-ionizing modality for the diagnosis of gastric lesions and a reasonable method for patients who need ongoing surveillance.

## Supporting information

S1 Table (XLSX)
